# Optimal Carbon Abatement Strategy for Manufacturers under Cap-and-Trade

**DOI:** 10.3390/ijerph191710987

**Published:** 2022-09-02

**Authors:** Qiangfei Chai, Yiming Li, Zhongdong Xiao, Kee-hung Lai

**Affiliations:** 1 School of Management, Nanjing University of Posts and Telecommunications, Nanjing 210023, China; 2 School of Economics and Management, Xidian University, Xi’an 710126, China; 3 School of Management, Xi’an Jiaotong University, Xi’an 710049, China; 4 Department of Logistics and Maritime Studies, The Hong Kong Polytechnic University, Hung Hom, Kowloon, Hong Kong, China

**Keywords:** sustainable operations, carbon emission abatement, remanufacturing, cap-and-trade

## Abstract

Carbon emission abatement is very important for manufacturers regulated by environmental policies. However, choosing an optimal carbon abatement strategy is difficult for many firms. This paper attempts to explore the appropriate carbon abatement strategy for firms that are regulated by cap-and-trade. Specifically, by bringing remanufacturing into consideration, this paper examines a manufacturer that has four alternative carbon abatement strategies: (1) do nothing, (2) invest in carbon abatement, (3) engage in remanufacturing, or (4) become involved in investment and remanufacturing together. The models of these four strategies are first developed in a monopolistic operating environment. The results show that among the four carbon abatement strategies, although the fourth strategy has the highest costs, it generates the largest profits for the manufacturer, passes the greatest benefits along to consumers, and has the best environmental performance. Next, this study is extended to a competitive environment. The results show that the optimal strategy in the monopolistic environment no longer maximizes profits, and decision guidance is offered for the manufacturer operating under such an environment.

## 1. Introduction

Environmental policies have transformed firms’ traditional operating environment characterized by unbound carbon emissions into a new situation in which polluters need to pay. For example, the largest carbon trading scheme in the world so far, the European Union Emission Trading Scheme (EU ETS), has operated for many years. Under EU ETS, firms are allocated certain allowances for carbon dioxide emission by the governing authorities. These firms have the right to sell their surplus allowances or purchase additional allowances in the carbon market, triggering their need to adjust operational strategy to comply with the scheme. For example, the Biofore Company (UPM), a magnate in the forest and paper industry in Finland, has been influenced by the EU ETS to invest over EUR 1 billion in renewable energy and carbon abatement, leading to 67% of fuels used at UPM being renewable energy and 78% of their electricity production having no carbon emissions [1]. The cap-and-trade policy is one of the most widely used environmental policies worldwide, and the EU ETS is an exemplary case of the cap-and-trade policy. Under the cap-and-trade policy, firms that emit more than their allowances need to pay more costs, while firms that emit less can earn more profits by selling surplus allowances. Therefore, it is necessary for firms to reduce their carbon emissions. However, how to choose an effective carbon abatement strategy to comply with environmental policies has not been addressed yet.

Investing in carbon abatement has been a popular operations strategy for carbon emission reduction (CER), which is widely adopted by many firms that are regulated by environmental policies. According to industry practice and academic research, such investment mainly includes using renewable energy as much as possible, upgrading production machines and using cleaner technologies, and reducing waste [2,3]. For example, Foxconn, an electronics contract manufacturer participating in the China Emissions Exchange, invested almost RMB 50 million in carbon abatement in 2013 (See http://www.tanpaifang.com/tanjiaoyianli/2016/0222/50786.html (accessed on 10 December 2020)). In addition, investment strategy is often studied in academic research. Zou et al. [4] studied a manufacturer’s financing and ordering decisions and a supplier’s pricing decisions with and without carbon abatement investment. Their focus was to analyze the influence of carbon abatement investment on the selection of financing equilibrium, not on choosing an optimal carbon abatement strategy. Zhu et al. [5] studied how to optimize the supply chain members’ investment strategy using evolutionary game theory. They showed that many firms still hesitate to invest in carbon abatement; however, they only studied the investment strategy. Turken et al. [6] investigated the influence of different environmental policies on investments in green technologies and end-of-pipe abatement technologies. They conducted an in-depth analysis of the investment strategy but did not explore other carbon abatement strategies. In fact, many studies are confined to investment decisions for CER, neglecting other possible strategies. Consequently, there is only decision guidance on the investment strategy for CER and no decision guidance on other strategies for CER.

It appears that investing in carbon abatement is not the only operations strategy option for firms to comply with environmental policies. As an environmentally friendly and profitable approach to organizing productive activities, remanufacturing has been developed for many years, with growing attention in many countries, including the USA, China, and Germany. Remanufacturing is a process in which used products can be restored to like-new condition. This approach has two performance advantages, namely, saving energy consumption with CER and saving production costs due to the utilization of used products or parts collected by firms to make products. According to the practices adopted by Caterpillar, a leading manufacturer of construction and mining equipment and one of the biggest remanufacturers in the world, remanufactured components can retain more than 85% of the energy required to manufacture new equipment. This company has reduced carbon emissions by over 1 million tons with remanufacturing practiced in the past 10 years (See https://www.caterpillar.com/en/news/caterpillarNews/governmental-affairs/multiple-lives-a-win-win-win.html (accessed on 19 December 2020)). Furthermore, remanufacturing can save 50% of production costs [7]. Due to these two aspects of performance benefits, a number of firms involved in remanufacturing attain better performance outcomes, including Rank Xerox, BMW, and IBM [8]. Compared with the investment strategy, the remanufacturing strategy not only can achieve CER but also can help manufacturers reduce production costs. These two beneficial aspects are significant factors for manufacturers in choosing a carbon abatement strategy. Therefore, the remanufacturing strategy should be explored as a carbon abatement strategy. However, most extant studies related to remanufacturing primarily focus on the cost-benefit and rarely incorporate the low-carbon character into their models. As a result, the potential of the remanufacturing strategy for CER under cap-and-trade has not been explored. In addition, a firm can employ both operations strategies, i.e., investing in carbon abatement and remanufacturing, for CER. However, this option is rarely stressed in extant studies. Caterpillar is an exemplary company employing both strategies to achieve sustainability.

This background leads us to ask the following questions. For a firm only making new products with their operations under cap-and-trade, which carbon abatement strategies (investment, remanufacturing, or both) should the firm choose? Second, how are the selling price, the production quantity, and the firm’s profits influenced by the choice of carbon abatement strategy? Will the firm’s optimal carbon abatement strategy vary between the monopolistic environment and the competitive environment?

This paper aims to provide guidelines for manufacturing firms operating under the cap-and-trade policy to make carbon abatement decisions. To investigate the impact of different carbon abatement strategies on firms’ profits and carbon emissions, this paper considers a monopoly that produces only new products and is regulated by cap-and-trade initially. The monopoly would be allocated certain allowances at the beginning of the period. She (This paper uses “she” to refer to the firm or the manufacturer throughout the paper) is free to purchase allowances to increase her production scale or to sell allowances at the expense of reducing production quantity. There are four strategies for the manufacturer to choose under the cap-and-trade policy: (1) do nothing, (2) invest in carbon abatement, (3) engage in remanufacturing, or (4) invest in carbon abatement and perform remanufacturing together. The four strategies is contrasted in terms of the selling price, the production quantity, the sustainability level, the product return rate, and the firm’s profits. Then, the analysis is extended to a competitive environment where a competitor produces substitutable products and is also regulated by cap-and-trade. These four strategies are also compared under this setting.

This paper makes the following contributions. Firstly, to the best of our knowledge, this study is the first to explore a manufacturer’s optimal carbon abatement strategy under cap-and-trade considering remanufacturing. Secondly, this paper finds that the remanufacturing strategy may perform better than the investment strategy, and even be the optimal carbon abatement strategy, when the cost savings of the remanufacturing strategy is higher than that of the investment strategy if the input is equal. Most studies related to manufacturers’ carbon abatement usually regard the investment strategy as the only approach for CER. However, this study shows that the remanufacturing strategy deserves to be considered by manufacturers for CER. Thirdly, from economic and environmental perspectives, this paper finds that the strategy involving both investment and remanufacturing is the optimal carbon abatement strategy in the monopolistic environment, but this strategy is not optimal in the competitive environment. Fourthly, this paper shows that the selection of optimal carbon abatement strategy in the competitive environment depends on the relationship between *SR_I_* and *SR_R_* (the quadratic cost savings per unit to input ratio); these ratios measure the efficiency of cost savings of carbon abatement strategies. Lastly, this paper finds that the ordering of the four strategies in terms of the optimal production quantity, the optimal sustainability level, and the optimal return rate does not vary between the monopolistic environment and the competitive environment.

The rest of the paper is organized as follows. Section 2 provides a brief literature review and identifies the contributions of the paper. Section 3 introduces the notations and related assumptions used in the paper. The models of different carbon abatement strategies in a monopolistic environment are developed, and the analytical results are presented in Section 4. Section 5 gives the comparison of different carbon abatement strategies in the monopolistic environment. Section 6 examines different carbon abatement strategies in a competitive environment. Finally, Section 7 concludes and offers directions for future research.

## 2. Literature Review

This paper is grounded in three streams of studies, including sustainable operations management considering environmental policy, carbon emission abatement, and remanufacturing. For the stream of sustainable operations management, this paper refers the reader to [9,10] for detailed reviews. A growing number of studies on production and operations management address environmental management issues. These studies incorporate the conventional operations management directions covering facility location [11,12], inventory management [13], supply chain coordination [14], pricing and production decisions [15,16], etc. Using simple models, Benjaafar et al. [17] show that CER is possible by adjusting operations strategy or collaborating with supply chain members, which is an alternative to investment in carbon abatement. Unlike these studies, this paper focuses on the optimal carbon abatement strategy selection rather than studying how to optimize operational decisions under the regulation of environmental policies.

Regarding CER, investment in carbon abatement has been a popular topic in extant studies on sustainability. Dong et al. [18] studied sustainability investment in decentralized and centralized supply chains under cap-and-trade and obtained optimal production quantity and investment in these two types of supply chains. Furthermore, they showed that the supply chain could achieve coordination through revenue-sharing contracts. Luo et al. [19] considered green technology investment in rival manufacturers’ decisions. They analyzed a pure competition model and a co-opetition model to investigate the role of the latter model in low-carbon manufacturing. Turken et al. [6] mainly focused on investigating the influence of different environmental regulations on investments in green technologies and end-of-pipe abatement technologies. However, all these studies considered only carbon abatement investment strategy for CER, neglecting other available carbon abatement strategies as important decisions for firms to make. Inspired by industrial practice, this paper introduces remanufacturing as the other carbon abatement strategy and considers carbon abatement as an important strategic choice for firms operating under cap-and-trade.

Regarding remanufacturing, there are numerous related publications. Some relevant and important studies are purposely selected to help readers better understand the position of this work. For readers looking for a more comprehensive literature review, Atasu et al. [20] and Rizova et al. [21] are recommended. Debo et al. [22] considered that different production technologies could restore different values from used products and investigated the production technology selection problem. Ferrer and Swaminathan [23] examined new and remanufactured product management problems in a monopoly environment with an extension to a duopoly environment in which a competitor utilizes the core component produced by the manufacturer to produce remanufactured products. These are earlier game theory studies addressing remanufacturing problems. Ferrer and Swaminathan [24] then examined the situation in which new and remanufactured products are distinguishable in the same environment. Chen et al. [25] studied collection strategies and pricing decisions of used products considering the competition between the online platform and traditional channels. From a market perspective, Chen and Chen [26] studied two types of typical recovered products in the Chinese market, remanufactured and refurbished products, and tried to find the equilibrium market structure. In addition, when considering remanufacturing, firms often face a situation where the demand, the time to collect used products, and the quality of used products are highly uncertain. Considering yield uncertainty, Niu et al. [27] investigated a retailer’s make-or-buy decision for remanufactured products under in-store competition between new and remanufactured products. Mutha et al. [28] analyzed a supplier’s optimal assortments of used products and a buyer’s optimal purchase quantity of various grades considering the different quality conditions of used products. Furthermore, there are numerous studies focusing on those operations challenges, including Zhao et al. [29], Zhao et al. [30], and Zhou et al. [31].

However, all these studies stress only the cost-benefit of remanufacturing, neglecting the low carbon character in their models. In addressing this research void, this paper studies both the low-cost advantage and the low carbon emission advantage of remanufacturing, with an extension to the new market situation where firms’ carbon emissions are restricted and in which remanufacturing may be a superior option to manufacturing. Specifically, this paper utilizes the low carbon emission advantage of remanufacturing for CER to maintain the firm’s profits because remanufacturing emits less pollution than producing new products, and thus, the firm does not need to reduce her production quantities to comply with environmental policies. This paper also utilizes the low-cost advantage of remanufacturing to reduce the firm’s production costs, which can indirectly increase the firm’s profits.

This study contributes knowledge to the literature in three aspects. First, this paper examines whether remanufacturing could be an effective carbon abatement strategy for firms that are regulated by cap-and-trade due to the low-cost advantage and the low carbon emission advantage of remanufacturing. Second, this paper studies both a monopolistic environment and a competitive environment considering cap-and-trade and examines whether optimal carbon abatement strategies vary between these two environments. Finally, this paper provides detailed decision guidance on how to choose an optimal carbon abatement strategy for manufacturers operating under cap-and-trade.

## 3. Model Assumptions and Notation

This paper considers a monopolistic manufacturer producing new products only and operating under cap-and-trade, with a subsequent extension to a competitive environment where there is a rival manufacturer making similar products as the manufacturer and operating under this policy. Let p denote the market price of the product. $c_{n}$ is the production cost per new product, e denotes the carbon emissions from production per new product, C denotes the carbon cap allocated to the manufacturer. Under cap-and-trade, the monopolist can purchase or sell carbon emission allowances. Therefore, let P denote the carbon price. T denotes the monopolist’s carbon trading quantity and it is positive when the monopolist buys carbon emission allowances and negative when the monopolist sells carbon emission allowances. For convenience of reference, the notation used in our model is summarized in Table 1.

The primary goal of this study is to investigate the impact of different carbon abatement strategies on firms’ performance (in terms of profits, selling price, and production quantity) and environmental performance (in terms of the sustainability level and the return rate of used products). Therefore, a downward linear demand function is used, q=Δ−p and $\Delta >c_{n}+Pe$. The downward linear demand function is a common demand function in extant studies [32,33]. The influence of the consumer’s environmental awareness on the demand function is not considered because this paper considers investments that can reduce carbon emissions without changing the characteristics of the product, such as using renewable energy and end-of-pipe technologies [34]. For simplicity, without loss of generality, this paper assumes the slope of the linear demand function is equal to 1 [33,35]. The demand function simplifies our modeling without influencing the comparison between different carbon abatement strategies. The condition for Δ is to ensure the demand is positive when the product is sold at cost.

It is assumed that there are no idle allowances left at the end of the period. This assumption means that the manufacturer either spends all her allowances on producing products or sells her surplus allowances at the end of the period to maximize her profits. The behavior is a tactical behavior (myopic behavior) rather than a strategic behavior (provident behavior). Manufacturers’ allowances management strategy warrants attention in future research. Our goal is to compare different carbon abatement strategies, and the manufacturers’ tactical behavior makes the analysis easier due to its tractability.

It is assumed that the investment in carbon abatement is a quadratic function of s, $I_{A}=Bs^{2}$, where B is a sustainability scaling coefficient and s represents the sustainability level of the product. The carbon emissions per product is e−βs after investment, where 0≤s≤e/β to ensure that the carbon emissions of producing a product are nonnegative, e is the carbon emissions from production per new product when the sustainability level equals 0, β is the sustainability coefficient that reflects the effect of sustainability level on reducing carbon emissions. This paper also assumes the collection effort is a quadratic function of τ, $I_{R}=A\tau^{2}$, where A is a collection scaling coefficient and τ represents the ratio of remanufactured products to all products made or the ratio of remanufactured parts to all parts contained in products, 0≤τ≤1. According to this assumption, the average production cost per product when the manufacturer chooses a remanufacturing strategy is $c_{r}=c_{n}\left(1-\tau \right)+\left(c_{n}-a\right)\tau =c_{n}-a\tau$, where a is the cost savings from production per remanufactured product. The average carbon emissions per product when the manufacturer chooses a remanufacturing strategy is $e_{r}=e\left(1-\tau \right)+\left(e-b\right)\tau =e-b\tau$, where b is the carbon emissions savings per remanufactured product.

Similar quadratic functions are widely used in extant studies, such as in the advertising field [36], investing in product quality improvement or the sustainability field [6,18], and the collection of used products in the closed-loop supply chain field [32,37]. Gurnani and Erkoc [38] characterized both the retailer’s selling effort and the manufacturer’s quality investment by similar quadratic functions. As this paper has similar considerations as the above studies, this paper refers to the collection effort function of Savaskan et al. [32]. The quadratic function can reflect the diminishing effect of the investment on demand or sustainability.

In addition, similar to Huang and Wang [39], Yang et al. [40], and Long et al. [41], this paper only considers used products that can be incorporated partially or wholly into a new product through remanufacturing, and thus, this paper assumes that the remanufactured products are treated as the new products in this paper. This kind of product exists in practice. For example, Fuji Xerox collects used copiers and incorporates used components into new copiers [42]. Kodak’s single-use cameras also contain remanufactured parts, and most consumers do not know it [43]. Moreover, HP sells remanufactured products “as new” and tries to make consumers believe that HP renewed products have no difference from HP new products [44]. This assumption makes the comparison of different carbon abatement strategies easier and more feasible.

Furthermore, it is assumed that the manufacturer’s decision horizon is limited to a single period. This paper assumes the manufacturer’s product has been introduced to the market for some time so that the manufacturer is able to collect adequate used products from consumers. Therefore, this single period can be seen as a steady-state period. For example, Caterpillar has been engaging in remanufacturing for many years and is able to collect millions of its used products every year. A single period allows us to easily compare the four carbon abatement strategies. A similar single-period assumption can be found in Hong et al. [45], Xue et al. [46], and Liu et al. [47].

## 4. Carbon Abatement Models under Cap-and-Trade

In this section, three carbon abatement models corresponding to the three carbon abatement strategies: the strategy of investing in carbon abatement (Model I), the remanufacturing strategy (Model R), and the strategy of becoming involved in carbon abatement investment and remanufacturing together (Model I & R) are developed. The scenario of the manufacturer doing nothing when facing cap-and-trade is used as the benchmark model (Model B). We compare the three carbon abatement models to the benchmark model with respect to the selling price, the production quantity, the sustainability level, the return rate, and the manufacturer’s profits.

### 4.1. Model B: The Benchmark Model

In the benchmark scenario, the manufacturer can only adjust her operations strategy to comply with the cap-and-trade policy, i.e., balancing production quantities and carbon emissions to maximize her profits. Therefore, the manufacturer optimizes:

(1) $$\underset{p}{Max}\Pi^{B}=\left(p-c_{n}\right)q-PT$$

s.t.eq=T+C

The above profit function is concave in p; thus, the optimal solution can be obtained by solving the first order condition of the profit function, shown as follows:

$$p^{*B}=\frac{\Delta +c_{n}+Pe}{2},q^{*B}=\frac{\Delta -c_{n}-Pe}{2}.$$

To understand the impact of cap-and-trade on the manufacturer’s decisions, the optimal decisions when the manufacturer makes products without cap-and-trade (Model W) is derived, shown as follows:

$$p^{*}=\frac{\Delta +c_{n}}{2},q^{*}=\frac{\Delta -c_{n}}{2}.$$

The optimal profits and carbon emissions of Model W and Model B can be obtained by substituting the optimal price and the optimal production quantity in the corresponding profit functions and carbon emissions functions. These results are listed in Table A1 in the Appendix A. The optimal price of Model B is higher than that of Model W, which means that the manufacturer being regulated by cap-and-trade would shift the additional cost arising from the policy to consumers for the purpose of making more profits. Correspondingly, the optimal production quantity of Model B is lower than that of Model W. Not surprisingly, the cap-and-trade policy restricts the manufacturer’s production positivity. However, it should be noted that the cap-and-trade policy is truly effective in reducing carbon emissions, as one can see in Table A1 in the Appendix A.

### 4.2. Model I: The Investment in Carbon Abatement Strategy

In most cases, firms invest in carbon abatement when they are regulated by cap-and-trade. Such investment mainly includes using renewable energy as much as possible, upgrading their production machines and technologies, and reducing waste. For example, Foxconn’s factory in China invested in upgrading their production line since they began participating in the China Emissions Exchange in 2013. Consequently, they saved many allowances and earned considerable profit from the strategy.

In this scenario, the manufacturer invests a certain amount of money ($I_{A}=Bs^{2}$) in carbon abatement so that the carbon emissions per product would reduce by βs, where β is the sustainability coefficient that reflects the effect of sustainability level on reducing carbon emissions. The carbon emissions per product is e−βs after investment, where 0≤s≤e/β to ensure that the carbon emissions of producing a product are nonnegative. Therefore, the manufacturer optimizes:

(2) $$\underset{p,s}{Max}\Pi^{I}=\left(p-c_{n}\right)q-PT-Bs^{2}$$

$$s.t.\left(e-\beta s\right)q=T+C$$

To ensure that the optimal solution of s is between 0 and e/β, we impose $\partial \Pi_{I}/{\left.\partial s\right|}_{s=e/\beta }\leq 0$, from which we can induce Assumption 1 (Shown in Section 4.3).

The manufacturer’s profit function is jointly concave in p and s, which is shown in the Appendix A (All proofs are shown in the Appendix A for the clarity of the paper.). Hence, the manufacturer’s optimal response can be derived from the first order conditions, shown as follows:

$$p^{*I}=\frac{2B\left(\Delta +c_{n}+Pe\right)-\Delta P^{2}\beta^{2}}{4B-P^{2}\beta^{2}},q^{*I}=\frac{2B\left(\Delta -c_{n}-Pe\right)}{4B-P^{2}\beta^{2}}\;\mathrm{and}\;s^{*I}=\frac{P\beta \left(\Delta -c_{n}-Pe\right)}{4B-P^{2}\beta^{2}}$$

The optimal profit and carbon emissions of Model I can be obtained by the substitution of $p^{*I}$ and $s^{*I}$, which are shown in Table A2 in the Appendix A.

### 4.3. Model R: The Remanufacturing Strategy

In this case, the manufacturer employs a remanufacturing strategy to abate her carbon emissions. The unique feature of this strategy lies in the merits of remanufacturing for CER and the production cost saving since it employs used products or core components in the production process. Thus, the carbon emissions per remanufactured product is e−b. The production cost per remanufactured product is $c_{n}-a$, and it is assumed that this cost includes the manufacturer’s collection cost and handling cost. As stated in Section 3, the average production cost per product of this strategy is $c_{r}=c_{n}\left(1-\tau \right)+\left(c_{n}-a\right)\tau =c_{n}-a\tau$. Thus, the manufacturer faces the problem:

(3) $$\underset{p,\tau }{Max}\Pi^{R}=\left(p-c_{n}+a\tau \right)q-PT-A\tau^{2}$$

$$s.t.\left(e-b\tau \right)q=T+C\;$$

Similar to Model I, to guarantee the optimal solution of τ is between 0 and 1, we impose the condition $\partial \Pi_{R}/{\left.\partial \tau \right|}_{\tau =1}\leq 0$. Consequently, we have Assumption 1 (See Lemma A1 in the Appendix A).

**Assumption** **1.** *The relationship between the sustainability scaling coefficient* B *and the collection scaling coefficient* A *is assumed to satisfy the condition* $B\geq AP^{2}\beta^{2}/\left(4A-\left(Pb+a\right)\left(\Delta -c_{n}-Pe\right)-{\left(Pb+a\right)}^{2}\right)$ *such that* $\tau^{*}\leq 1$ *and* $s^{*}\leq e/\beta$ *exist in all scenarios.*

Again, the manufacturer’s profit function is proved to be jointly concave in p and τ. The manufacturer’s optimal response in this model can be obtained by solving the first order conditions and is shown as follows:

$$p^{*R}=\frac{2A\left(\Delta +c_{n}+Pe\right)-\Delta {\left(Pb+a\right)}^{2}}{4A-{\left(Pb+a\right)}^{2}},q^{*R}=\frac{2A\left(\Delta -c_{n}-Pe\right)}{4A-{\left(Pb+a\right)}^{2}}\;\mathrm{and}\;\tau^{*R}=\frac{\left(Pb+a\right)\left(\Delta -c_{n}-Pe\right)}{4A-{\left(Pb+a\right)}^{2}}$$

Substituting the above optimal values in the manufacturer’s profit function and the carbon emissions function, we have the manufacturer’s optimal profit and carbon emissions, shown in Table A2 in the Appendix A.

### 4.4. Model I&R: The Strategy Involving Both Investment and Remanufacturing

This strategy combines the respective advantages of the investment in carbon abatement strategy and the remanufacturing strategy and thus has twice the advantages in carbon abatement. However, employing both strategies means the manufacturer needs more inputs. Therefore, the manufacturer has to balance the trade-off. An example of this strategy is Caterpillar’s sustainability strategy; Caterpillar not only engages in remanufacturing but also invests in renewable energy technologies. As a result, Caterpillar reduced total absolute carbon emissions by 29% from 2006 to 2016 (See https://www.caterpillar.com/en/company/sustainability/sustainability-report.html (accessed on 16 July 2021)). In this model, the manufacturer faces the problem:

(4) $$\underset{p,\tau ,s}{Max}\Pi^{I\&R}=\left(p-c_{n}+a\tau \right)q-PT-A\tau^{2}-Bs^{2}$$

$$s.t.\left(e-b\tau -\beta s\right)q=T+C\;$$

To ensure the optimal solution of τ is between 0 and 1 and the optimal solution of s is between 0 and e/β, we impose the conditions of $\partial \Pi_{I\&R}/{\left.\partial \tau \right|}_{\tau =1}\leq 0$ and $\partial \Pi_{I\&R}/{\left.\partial s\right|}_{s=e/\beta }\leq 0$. Similarly, we have the former Assumption 1.

The profit function of the model is jointly concave in p, τ, and s. Therefore, the optimal response of the manufacturer can be obtained by solving the first order conditions, shown as follows:

$$p^{*I\&R}=\frac{2AB\left(\Delta +c_{n}+Pe\right)-\Delta B{\left(Pb+a\right)}^{2}-\Delta AP^{2}\beta^{2}}{A\left(4B-P^{2}\beta^{2}\right)-B{\left(Pb+a\right)}^{2}},q^{*I\&R}=\frac{2AB\left(\Delta -c_{n}-Pe\right)}{A\left(4B-P^{2}\beta^{2}\right)-B{\left(Pb+a\right)}^{2}}$$

$$s^{*I\&R}=\frac{AP\beta \left(\Delta -c_{n}-Pe\right)}{A\left(4B-P^{2}\beta^{2}\right)-B{\left(Pb+a\right)}^{2}},\tau^{*I\&R}=\frac{B\left(Pb+a\right)\left(\Delta -c_{n}-Pe\right)}{A\left(4B-P^{2}\beta^{2}\right)-B{\left(Pb+a\right)}^{2}}\;$$

Substituting the above optimal values in the manufacturer’s profit function and the carbon emissions function, we have the manufacturer’s optimal profit and carbon emissions, shown in Table A2 in the Appendix A.

## 5. Comparison of Different Strategies

After formulating different models corresponding to different strategies in Section 4, the four models are compared in this section. Based on the results of the four models shown in Table A1 and Table A2 in the Appendix A, some interesting propositions can be obtained.

**Proposition** **1.** *The production quantity, the selling price, and the carbon emissions are affected by the carbon price and carbon emissions of the product, while they are not affected by the cap in Model B. However, the cap is a significant factor that influences the manufacturer’s profits. More specifically, when* $4C>e\left(2\Delta -2c_{n}-Pe\right)$ *(or equivalently* $T<-Pe^{2}/4$ *), cap-and-trade has a favorable effect on the manufacturer, which means that the manufacturer can make more profits under cap-and-trade.*

Note that Proposition 1 shows that cap-and-trade is beneficial for the manufacturer only on the premise that the cap allocated to the manufacturer is sufficiently high such that the manufacturer can retain certain allowances to sell in the carbon market after production (T<0). However, this condition is hard to achieve in the later stage of cap-and-trade policy. For example, the EU ETS aims for CER by 20% relative to 1990s emission levels before 2020 through three phases. The participating firms received adequate allowances that were sufficient to cover their emissions in the first phase; however, the cap was tightened in the second phase and further reduced each year by 1.74% in the last phase [15]. Once the cap decreases lower than the threshold, the manufacturer would be negatively impacted by cap-and-trade if it does not pursue any strategies to mitigate its carbon emissions.

**Proposition** **2.** *The optimal production quantities of the four models are related as* $q^{*I\&R}>q^{*R},q^{*I}>q^{*B}$ *. Correspondingly, the optimal selling prices are related as* $p^{*I\&R}<p^{*R},p^{*I}<p^{*B}$ *. The relationship between* $q^{*I}$ *and* $q^{*R}$ *, as well as* $p^{*I}$ *and* $p^{*R}$ *, are characterized by the relationship between* ${\left(Pb+a\right)}^{2}/A$ *and* $P^{2}\beta^{2}/B$ *. Specifically, when* ${\left(Pb+a\right)}^{2}/A>P^{2}\beta^{2}/B$ *, we have* $q^{*R}>q^{*I}$ *and* $p^{*R}<p^{*I}$ *; otherwise,* $q^{*R}<q^{*I}$ *and* $p^{*R}<p^{*I}$.

The optimal production quantity of the Model B is the lowest because of the influence of cap-and-trade. The optimal selling price in Model B is the highest among the four strategies, which means that the manufacturer would shift the burden of cap-and-trade to consumers. However, the optimal production quantity would increase if the manufacturer employed any strategies. The rationale is that employing a carbon abatement strategy would reduce the carbon emissions cost, and this benefit would be passed on to consumers by reducing the selling price. As a result, the demand would increase. Employing both investments in carbon abatement and remanufacturing strategies leads to the highest benefits for consumers, as the selling price is the lowest among the four strategies. Accordingly, the demand is highest among the four strategies.

It is interesting to note the relationship between the selling price of Model I and Model R. ${\left(Pb+a\right)}^{2}/A$ and $P^{2}\beta^{2}/B$ can be seen as the quadratic cost savings per unit to input ratio (for simplicity, let *SR* denote this ratio, $SR_{R}={\left(Pb+a\right)}^{2}/A$) and $SR_{I}=P^{2}\beta^{2}/B$ of strategy R and I, respectively. Because $\left(Pb+a\right)\tau$ and Pβs are the cost savings per unit of strategy R and I, respectively; $A\tau^{2}$ is the collection effort of strategy R and $Bs^{2}$ is the investment of strategy I, respectively. This ratio measures the efficiency of cost savings of a carbon abatement strategy and is useful for evaluating two different strategies. As Proposition 2 shows, when *SR_R_* is higher than *SR_I_*, which means that the cost savings of strategy R is higher than strategy I when the input is equal, the strategy R can generate more benefits for consumers, which can be reflected in the selling price.

**Proposition** **3.** *The optimal sustainability levels of Model I and Model I&R are related as*$s^{*I\&R}>s^{*I}$*. The optimal return rates of used products of Model R and Model I&R are related as*$\tau^{*I\&R}>\tau^{*R}$.

Proposition 3 shows that Model I&R has the best environmental performance among Model I, Model R, and Model I&R. Surprisingly, contrary to our conventional thought that employing two strategies together requires a lower sustainability level and collection level, this finding highlights that employing both investments in carbon abatement and remanufacturing strategies is a better option for the manufacturer from the perspective of environmental protection.

**Proposition** **4.** *The manufacturer’s optimal profits for different strategies are related as* $\Pi^{*I\&R}>\Pi^{*R},\Pi^{*I}>\Pi^{*B}$*. The relationship between* $\Pi^{*R}$ *and* $\Pi^{*I}$ *are characterized by the relationship between* ${\left(Pb+a\right)}^{2}/A$ *and* $P^{2}\beta^{2}/B$ *. Specifically, when* ${\left(Pb+a\right)}^{2}/A>P^{2}\beta^{2}/B$ *, we have* $\Pi^{*R}>\Pi^{*I}$ *; otherwise,* $\Pi^{*R}<\Pi^{*I}$.

Similar to the relationship between the optimal production quantities of different models, Proposition 4 demonstrates that employing both strategies can generate the highest profits among the four strategies, while the benchmark strategy has the worst economic performance. It is better for firms that are regulated by cap-and-trade to adopt at least one strategy from investment in carbon abatement or remanufacturing, and if it is possible, firms are advised to employ both strategies together. For firms seeking to know how to balance the trade-off between investment in carbon abatement strategy and remanufacturing strategy, this paper also presents the condition under which investment in carbon abatement strategy is better or remanufacturing strategy is better. It is interesting to note that employing both strategies has not only the best environmental performance but also the best economic performance.

## 6. Extension: Competitive Environment

In most cases, firms may face competition from other firms that make similar products. For example, in the automotive industry, each firm faces fierce competition in selling its cars. Thus, competitive models are developed in this section. It is assumed that there is a competitor regulated by cap-and-trade in the market making similar products as the manufacturer, and these two types of products are interchangeable. Because this paper focuses on the comparison between different carbon abatement strategies, it is assumed that the carbon cap allocated to the manufacturer and the competitor are the same and that the carbon emissions from producing each new product are the same when the manufacturer does not exert any of these carbon abatement strategies (the models can be easily extended to a situation in which the carbon cap and carbon emissions are different). We adopt similar demand functions as are used in the Bertrand Model, defined as follows:

$$q_{1}=\alpha \Delta -p_{1}+d_{1}p_{2}$$

$$q_{2}=\left(1-\alpha \right)\Delta -p_{2}+d_{2}p_{1}\;$$

where $q_{1}$ represents the manufacturer’s demand and $q_{2}$ represents the competitor’s demand. The parameter α denotes the degree of customer loyalty to the manufacturer or the brand influence of the manufacturer, and 1−α denotes the degree of customer loyalty to the competitor or the brand influence of the competitor, 0≤α≤1. 8$d_{1}$ and $d_{2}$ are cross-price sensitivity parameters to the competitor’s product and to the manufacturer’s product, respectively. This paper assumes the parameters $d_{1}$ and $d_{2}$ satisfy $0\leq d_{1},d_{2}\leq 1$, which means that the influence of the manufacturer’s (the competitor’s) own price on her own demand is larger than the influence of her competitor’s price on the demand.

### 6.1. Model CB: The Benchmark Model in a Competitive Environment

In this model, the manufacturer does not use any carbon abatement strategies. Therefore, the manufacturer’s objective function is:

(5) $$\underset{p_{1}}{Max}\Pi_{M}^{CB}=\left(p_{1}-c_{n}\right)q_{1}-PT_{1}$$

$$s.t.\;eq_{1}=T_{1}+C.$$

The competitor’s objective function is:

(6) $$\underset{p_{2}}{Max}\Pi_{C}^{CB}=\left(p_{2}-c_{c}\right)q_{2}-PT_{2}$$

$$s.t.\;eq_{2}=T_{2}+C.$$

The above two profit functions are concave in $p_{1}$ and $p_{2}$; thus, the manufacturer’s optimal response can be obtained by simultaneously solving the first order conditions, shown as follows:

$$\;p_{1}^{*CB}=-\frac{2\left(\alpha \Delta +c_{n}+Pe\right)+d_{1}\left(\left(1-\alpha \right)\Delta +c_{c}+Pe\right)}{d_{1}d_{2}-4},$$

$$q_{1}^{*CB}=\frac{-d_{1}\left(\left(1-\alpha \right)\Delta +c_{c}+Pe\right)+\left(2-d_{1}d_{2}\right)\left(Pe+c_{n}\right)-2\alpha \Delta }{d_{1}d_{2}-4}.$$

The manufacturer’s optimal profit in this model can be obtained by substitution of the above two optimal solutions, denoted by $\Pi_{M}^{*CB}$.

### 6.2. Model CI: The Investment Strategy in a Competitive Environment

In this scenario, the manufacturer adopts the investment strategy to reduce carbon emissions for the purpose of obtaining low carbon advantages under cap-and-trade as well as protecting the environment. Similar to the monopolistic environment, the manufacturer invests $I_{A}=Bs^{2}$ such that the carbon emission for producing a product can reduce βs.

Therefore, the manufacturer’s objective function is:

(7) $$\underset{p_{1},s}{Max}\Pi_{M}^{CI}=\left(p_{1}-c_{n}\right)q_{1}-PT_{1}-Bs^{2}$$

$$s.t.\;\left(e-\beta s\right)q_{1}=T_{1}+C.$$

The competitor’s objective function is the same as (6). The manufacturer’s and the competitor’s profit functions are concave in *p*_1_, *p*_2_, and *s*. Therefore, the first order conditions characterize the manufacturer’s optimal solutions, shown as follows:

$$p_{1}^{*CI}=\frac{d_{1}\left(2B-P^{2}\beta^{2}\right)\left(\left(1-\alpha \right)\Delta +c_{c}+Pe\right)+4B\left(\alpha \Delta +c_{n}+Pe\right)-2\alpha \Delta P^{2}\beta^{2}}{P^{2}\beta^{2}\left(d_{1}d_{2}-2\right)-2B\left(d_{1}d_{2}-4\right)},$$

$$q_{1}^{*CB}=\frac{2B\left(d_{1}\left(\left(1-\alpha \right)\Delta +c_{c}+Pe\right)+\left(d_{1}d_{2}-2\right)\left(Pe+c_{n}\right)+2\alpha \Delta \right)}{P^{2}\beta^{2}\left(d_{1}d_{2}-2\right)-2B\left(d_{1}d_{2}-4\right)},$$

$$s^{*CB}=\frac{P\beta \left(d_{1}\left(\left(1-\alpha \right)\Delta +c_{c}+Pe\right)+\left(d_{1}d_{2}-2\right)\left(Pe+c_{n}\right)+2\alpha \Delta \right)}{P^{2}\beta^{2}\left(d_{1}d_{2}-2\right)-2B\left(d_{1}d_{2}-4\right)}.$$

The manufacturer’s optimal profit in this model can be obtained by substitution of the above three optimal solutions, denoted by $\Pi_{M}^{*CI}$.

### 6.3. Model CR: The Remanufacturing Strategy in a Competitive Environment

In this model, the manufacturer engages in remanufacturing to obtain both a low carbon emission advantage and a low production cost advantage. It has been proved that remanufacturing is an effective marketing strategy under competition when there is no cap-and-trade regulation [20]. However, the effectiveness of remanufacturing under cap-and-trade remains unclear. We speculate that remanufacturing is also an effective competitive strategy under competition when the manufacturer is regulated by cap-and-trade because the manufacturer can obtain a low carbon emission advantage through remanufacturing.

The manufacturer’s objective function is:

(8) $$\underset{p_{1},\tau }{Max}\Pi_{M}^{CR}=\left(p_{1}-c_{n}+a\tau \right)q_{1}-PT_{1}-A\tau^{2}$$

$$s.t.\;\left(e-b\tau \right)q_{1}=T_{1}+C.$$

The competitor’s objective function is the same as (6). The manufacturer’s and the competitor’s profit functions are concave in $p_{1}$, $p_{2}$, and τ. Therefore, solving the first order conditions, the manufacturer’s optimal solutions can be obtained, shown as follows:

$$p_{1}^{*CR}=\frac{d_{1}\left(2A-{\left(Pb+a\right)}^{2}\right)\left(\left(1-\alpha \right)\Delta +c_{c}+Pe\right)+4A\left(\alpha \Delta +c_{n}+Pe\right)-2\alpha \Delta {\left(Pb+a\right)}^{2}}{\left(d_{1}d_{2}-2\right){\left(Pb+a\right)}^{2}-2A\left(d_{1}d_{2}-4\right)},$$

$$q_{1}^{*CR}=\frac{2A\left(d_{1}\left(\left(1-\alpha \right)\Delta +c_{c}+Pe\right)+\left(d_{1}d_{2}-2\right)\left(Pe+c_{n}\right)+2\alpha \Delta \right)}{\left(d_{1}d_{2}-2\right){\left(Pb+a\right)}^{2}-2A\left(d_{1}d_{2}-4\right)},$$

$$\tau^{*CR}=\frac{\left(Pb+a\right)\left(d_{1}\left(\left(1-\alpha \right)\Delta +c_{c}+Pe\right)+\left(d_{1}d_{2}-2\right)\left(Pe+c_{n}\right)+2\alpha \Delta \right)}{\left(d_{1}d_{2}-2\right){\left(Pb+a\right)}^{2}-2A\left(d_{1}d_{2}-4\right)}.$$

The manufacturer’s optimal profit in this model can be obtained by substitution of the above three optimal solutions, denoted by $\Pi_{M}^{*CR}$.

### 6.4. Model CI&CR: Both Strategies in a Competitive Environment

The manufacturer adopts both the investment strategy and the remanufacturing strategy in this model. Therefore, the manufacturer has to pay the cost of investment and remanufacturing. However, she can obtain the lowest carbon emissions for production per product and the lowest production cost advantages accordingly.

The manufacturer’s objective function is:

(9) $$\underset{p_{1},s,\tau }{Max}\Pi_{M}^{CI\&CR}=\left(p_{1}-c_{n}+a\tau \right)q_{1}-PT_{1}-A\tau^{2}-Bs^{2}$$

$$s.t.\;\left(e-b\tau -\beta s\right)q_{1}=T_{1}+C.$$

The competitor’s objective function is the same as (6). The manufacturer’s profit function is concave in *p*_1_, *s*, and *τ*. The competitor’s profit function is concave in *p*_2_. Therefore, the manufacturer’s optimal solutions can be obtained by solving the first order conditions, shown as follows:

$$p_{1}^{*CI\&CR}=\frac{d_{1}\left(A\left(2B-P^{2}\beta^{2}\right)-B{\left(Pb+a\right)}^{2}\right)\left(\left(1-\alpha \right)\Delta +c_{c}+Pe\right)+4AB\left(\alpha \Delta +c_{n}+Pe\right)}{\left(d_{1}d_{2}-2\right)\left(B{\left(Pb+a\right)}^{2}+AP^{2}\beta^{2}\right)-2AB\left(d_{1}d_{2}-4\right)},\;\;\;-\frac{2\alpha \Delta \left(B{\left(Pb+a\right)}^{2}+AP^{2}\beta^{2}\right)}{\left(d_{1}d_{2}-2\right)\left(B{\left(Pb+a\right)}^{2}+AP^{2}\beta^{2}\right)-2AB\left(d_{1}d_{2}-4\right)}$$

$$q_{1}^{*CI\&CR}=\frac{2AB\left(d_{1}\left(\left(1-\alpha \right)\Delta +c_{c}+Pe\right)+\left(d_{1}d_{2}-2\right)\left(Pe+c_{n}\right)+2\alpha \Delta \right)}{\left(d_{1}d_{2}-2\right)\left(B{\left(Pb+a\right)}^{2}+AP^{2}\beta^{2}\right)-2AB\left(d_{1}d_{2}-4\right)},$$

$$s^{*CI\&CR}=\frac{P\beta A\left(d_{1}\left(\left(1-\alpha \right)\Delta +c_{c}+Pe\right)+\left(d_{1}d_{2}-2\right)\left(Pe+c_{n}\right)+2\alpha \Delta \right)}{\left(d_{1}d_{2}-2\right)\left(B{\left(Pb+a\right)}^{2}+AP^{2}\beta^{2}\right)-2AB\left(d_{1}d_{2}-4\right)},$$

$$\tau^{*CI\&CR}=\frac{\left(Pb+a\right)\left(d_{1}\left(\left(1-\alpha \right)\Delta +c_{c}+Pe\right)+\left(d_{1}d_{2}-2\right)\left(Pe+c_{n}\right)+2\alpha \Delta \right)}{\left(d_{1}d_{2}-2\right)\left(B{\left(Pb+a\right)}^{2}+AP^{2}\beta^{2}\right)-2AB\left(d_{1}d_{2}-4\right)}.$$

The manufacturer’s optimal profit in this model can be obtained by substitution of the above four optimal solutions, denoted by $\Pi_{M}^{*CI\&CR}$.

### 6.5. Comparison of Different Strategies under Competition

In the competitive environment, by comparing different strategies, some interesting propositions are obtained.

**Proposition** **5.** *The optimal production quantities of the four models in a competitive environment are related as*$q^{*CI\&CR}>q^{*CR},q^{*CI}>q^{*CB}$*. The relationship between*$q^{*CI}$*and*$q^{*CR}$*is characterized by the relationship between*${\left(Pb+a\right)}^{2}/A$*and*$P^{2}\beta^{2}/B$*. Specifically, when*${\left(Pb+a\right)}^{2}/A>P^{2}\beta^{2}/B$*, we have*$q^{*CR}>q^{*CI}$*; otherwise,*$q^{*CR}<q^{*CI}$.

Adopting any one of the carbon abatement strategies in a competitive environment can effectively reduce carbon emissions per new product and carbon emissions costs and thus indirectly increases the manufacturer’s production capacity. The competitor’s production capacity is restricted by cap-and-trade, and thus the competitor tends to increase its price or reduce its production quantity, either of which would increase the demand for the manufacturer’s product. The demand for the manufacturer’s product is largest when the manufacturer employs both strategies (the CI&CR strategy). The impact of the investment strategy and the remanufacturing strategy on the manufacturer’s optimal production quantity is uncertain. When *SR_R_* is higher than *SR_I_*, which means that the cost savings of the remanufacturing strategy are higher than that of the investment strategy when the input is equal, employing the remanufacturing strategy is more economical and can lead to greater demand. However, when *SR_R_* is lower than *SR_I_*, employing the investment strategy is a wise choice. Surprisingly, the impact of the four strategies on the manufacturer’s optimal production quantities in the competitive environment is the same as the impact in the monopolistic environment, which means that the ordering of the four strategies does not vary between these two environments.

**Proposition** **6.** *The optimal sustainability levels of Model CI and Model CI&CR are related as*$s^{*CI\&CR}>s^{*CI}$*. The optimal return rates of used products in Model CR and Model CI&CR are related as*$\tau^{*CI\&CR}>\tau^{*CR}$.

Surprisingly, the impact of the CI (CR) strategy and the CI&CR strategy on the manufacturer’s optimal sustainability level (return rate) in the competitive environment is the same as the impact in the monopolistic environment. Proposition 6 shows that the CI&CR strategy has the best environmental performance either on the optimal sustainability level or on the optimal return rate. The ordering of environmental performance of the four strategies does not vary between the monopolistic environment and the competitive environment.

**Proposition** **7.** *The manufacturer’s optimal profit from different strategies under the competitive environment are related as follows:*

*(i)*  *If*$SR_{I}<4-m$*,*$\Pi_{M}^{*CI}>\Pi_{M}^{*CR}$.
*(ii)*  *If*$SR_{R}<4-m$*,*$\Pi_{M}^{*CR}>\Pi_{M}^{*CB}$.
*(iii)*  *When*$SR_{R}<SR_{I}$*, if*$SR_{R}>4-4m/\left(4-SR_{I}\right)$*,*$\Pi_{M}^{*CR}>\Pi_{M}^{*CI}$
  *When*$SR_{R}>SR_{I}$*, if*$SR_{R}<4-4m/\left(4-SR_{I}\right)$*,*$\Pi_{M}^{*CR}>\Pi_{M}^{*CI}$.
*(iv)*  *If*$SR_{I}+SR_{R}<\min\left\{4-4m/\left(4-SR_{I}\right),4-4m/\left(4-SR_{R}\right)\right\}$*,* $\Pi_{M}^{*CI\&CR}>\Pi_{M}^{*CR},\Pi_{M}^{*CI}>\Pi_{M}^{*CB}$

*where*$SR_{I}=P^{2}\beta^{2}/B$*,*$SR_{R}={\left(Pb+a\right)}^{2}/A$*,*$m=d_{1}^{2}d_{2}^{2}/{\left(d_{1}d_{2}-2\right)}^{2}$.

**Figure 1:**
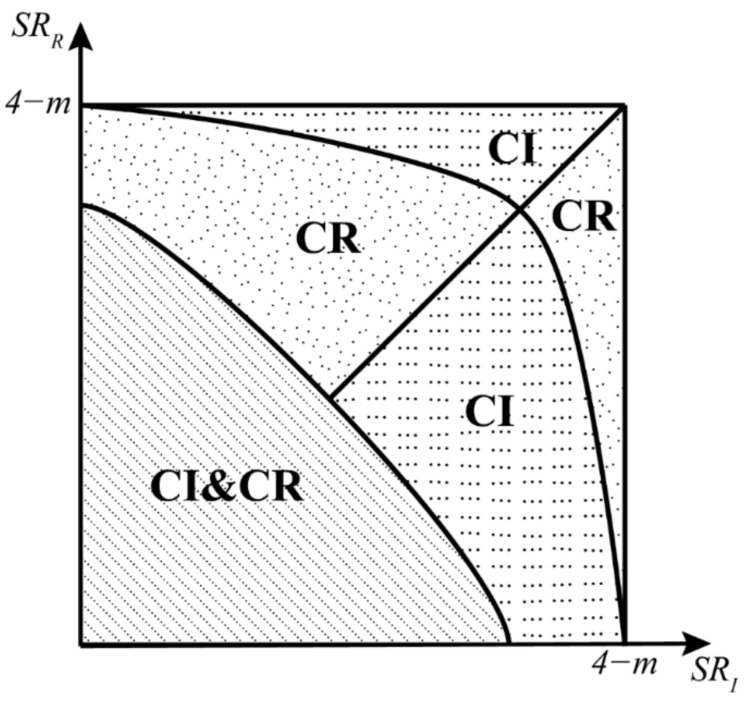
Which carbon abatement strategy is optimal? Note. $SR_{I}=P^{2}\beta^{2}/B$ and $SR_{R}={\left(Pb+a\right)}^{2}/A$ denote the quadratic cost savings per unit to input ratio of strategy I and R, respectively (defined in Section 5). These ratios measure the efficiency of cost savings of a carbon abatement strategy and are useful for evaluating different strategies.

Proposition 7 implies that the ordering of the manufacturer’s profit of different carbon abatement strategies is uncertain in a competitive environment, which is contrary to the monopolistic environment. Figure 1 shows the manufacturer’s decision zones in the competitive environment. It is surprising that both the investment strategy and the remanufacturing strategy are no longer optimal in the competitive environment. This is because the competitor captures a part of the market, and thus the manufacturer’s market share decreases. As a result, the manufacturer’s profit is affected. Therefore, under certain conditions, when *SR_R_* is larger than *SR_I_* ($SR_{R}+4m/\left(4-SR_{R}\right)>4-SR_{I}$), employing both strategies costs more than employing only the remanufacturing strategy, and it is optimal to employ only the remanufacturing strategy rather than both strategies. Similarly, under certain conditions, when *SR_I_* is larger than *SR_R_* ($SR_{I}+4m/\left(4-SR_{I}\right)>4-SR_{R}$), employing the investment strategy is optimal. It means that managers should care about *SR_I_* and *SR_R_* (the quadratic cost savings per unit to input ratio) when choosing an optimal carbon abatement strategy. Both higher *SR_I_* and *SR_R_* cannot guarantee that both strategies are optimal, and the critical conditions that help managers to make decisions shown in Proposition 7 should be noted.

## 7. Conclusions

With the implementation of the cap-and-trade policy for the purpose of CER and sustainable development by many countries, firms have to put their carbon emissions into their management agenda. However, investments in carbon abatement, such as investment in renewable energy, upgrading production machines to save energy, or investment in end-of-pipe technologies, are usually considered to be the exclusive approach for CER. Remanufacturing, an environmentally friendly and profitable production approach, is always neglected when firms are making decisions or researchers are conducting studies on CER. Choosing appropriate carbon abatement strategies has an important influence on firms, governments, and consumers. Accordingly, this paper incorporates a remanufacturing strategy as one of the important carbon abatement strategies and identifies the optimal carbon abatement strategy in a monopolistic and competitive environment. In addition, this paper also investigates the manufacturer’s optimal decisions under different strategies in different market environments.

In the monopolistic environment, this study finds the following conclusions. Firstly, the manufacturer would shift the additional cost arising from the cap-and-trade policy to consumers, and this policy restricts the manufacturer’s production positivity. However, the cap-and-trade policy is truly effective in reducing carbon emissions. Secondly, cap-and-trade may be beneficial to the manufacturer only when the carbon cap is sufficiently large, which is hard to achieve due to regulators’ purpose for CER. Thirdly, the manufacturer should adopt at least one carbon abatement strategy because both the economic and environmental performance of employing any one of the carbon abatement strategies is better than the economic and environmental performance of doing nothing. Fourthly, when *SR_R_* is higher than *SR_I_*, which means that the cost savings of the remanufacturing strategy is higher than that of the investment in carbon abatement strategy when the input is equal, the remanufacturing strategy can generate more benefits for consumers and generate more profits for the manufacturer. Lastly, among the three carbon abatement strategies, employing investment in carbon abatement and remanufacturing together generates the largest profits for the manufacturer and passes on the greatest benefits to consumers through reducing product prices. In addition, this strategy can increase the product’s sustainability level and used product return rate from an environmental perspective.

In the competitive environment, this study finds the following conclusions. Firstly, it is interesting to note that the ordering of the four strategies in terms of the optimal production quantity, the optimal sustainability level, and the optimal return rate is the same as the monopolistic environment. Secondly, the CI&CR strategy has the best environmental performance both on the optimal sustainability level and on the optimal return rate. Thirdly, it is surprising that the CI&CR strategy is no longer always the optimal carbon abatement strategy from an economic perspective in this competitive environment due to the impact of competition. Fourthly, managers should pay attention to the quadratic cost savings per unit to input ratio *SR_I_* and *SR_R_* when choosing an optimal carbon abatement strategy because these ratios measure the efficiency of cost savings of carbon abatement strategies. Under some conditions, employing the CI&CR strategy costs more than employing only the remanufacturing strategy or only the investment strategy, and it is optimal to employ only the remanufacturing strategy or only the investment strategy rather than the CI&CR strategy. Both higher *SR_I_* and *SR_R_* do not mean the CI&CR strategy is optimal. Section 6.5 provides detailed guidance on decisions in this environment for the manufacturer.

There are several avenues for future research to extend our study. First, this paper made several assumptions that can be relaxed to obtain more comprehensive insights into carbon abatement strategies. This paper assumes that the infrastructure for the remanufacturing equipment already exists such that the manufacturer has no barriers to remanufacturing. Based on the analysis, we speculate that introducing such an infrastructure would make the performance of the investment strategy better than the remanufacturing strategy when the cost savings and carbon emissions savings from remanufacturing are low. However, the performance rankings (in terms of the optimal profit, the optimal production quantity, the optimal sustainability level, and the optimal return rate) of becoming involved in both investment and remanufacturing strategies among the three strategies are not affected. This paper only studied the single-period game, and the manufacturer has no idle allowances left at the end of the period; an extension of a multi-period game can explore the manufacturer’s behavior in managing the resource of allowances. Second, this paper focuses on used products that can be incorporated partially or wholly into a new product through remanufacturing. One interesting extension is to study the product when the new product and the remanufactured product are different. Finally, this paper does not consider the influence of the product’s sustainability level or low carbon characteristics on demand during the competitive process, and this direction warrants future research attention.

## Figures and Tables

**Table 1:** List of Notations.

Notation	Definition
p,q	The price and production quantities of the new product, respectively
s	The sustainability level of the product
τ	The return rate of used products
Δ	Size of the potential product market
$c_{n}$	The production cost per new product
P	The carbon price in the carbon trading market
e	The carbon emissions from production per new product
C	The carbon cap allocated to the manufacturer
T	The carbon trading quantity. T>0 represents the manufacturer buys carbon emission allowances from a carbon trading market; T<0 represents the manufacturer sells carbon emission allowances to a carbon trading market
a	The cost savings from production per remanufactured product com-pared to production per new product
b	The carbon emissions savings per remanufactured product
A,B	The collection scaling coefficient and the sustainability scaling coeffi-cient, respectively
β	The sustainability coefficient that reflects the effect of sustainability level on reducing carbon emissions
α	The degree of customer loyalty to the manufacturer
$d_{1},\;d_{2}$	The cross-price sensitivity parameters to the competitor’s product and to the manufacturer’s product, respectively

## Data Availability

Not applicable.

## Appendix A

**Lemma** **A1.** *If the condition of* $\partial \Pi_{I\&R}/{\left.\partial \tau \right|}_{\tau =1}\leq 0$ *is satisfied, then* $s^{*I}$*,* $s^{*I\&R}$ *are all between 0 and* e/β. $\tau^{*R}$*,* $\tau^{*I\&R}$ *are all between 0 and 1.*

**Proof of Lemma** **A1.** (1) Consider *Model I: The investment in carbon abatement strategy* For $\Pi_{I}$ to be concave in p and s*,* $\Pi_{I}$ is supposed to satisfy the following conditions:

(i) $\partial^{2}\Pi_{I}/\partial p^{2}<0$
(ii) $\partial^{2}\Pi_{I}/\partial s^{2}<0$

The second order principal minor of the Hessian matrix of $\Pi_{I}$ should be positive, which is $D_{2}\left(p,s\right)=\left|\begin{array}{cc} \partial^{2}\Pi_{I}/\partial p^{2} & \partial^{2}\Pi_{I}/\partial p\partial s\\ \partial^{2}\Pi_{I}/\partial s\partial p & \partial^{2}\Pi_{I}/\partial s^{2} \end{array}\right|=\left|\begin{array}{cc} -2 & -P\beta \\ -P\beta & -2B \end{array}\right|=4B-P^{2}\beta^{2}>0$. (i) and (ii) hold because $\partial^{2}\Pi_{I}/\partial p^{2}=-2<0$ and $\partial^{2}\Pi_{I}/\partial s^{2}=-2B<0$, since B>0. To make sure the optimal solution of s is between 0 and e/β, we impose the condition $\partial \Pi_{I}/{\left.\partial s\right|}_{s=e/\beta }\leq 0$, from which we can obtain the result

(A1) $$B\geq P\beta^{2}\left(\Delta -c_{n}\right)/4e$$

Therefore, we have $D_{2}\left(p,s\right)\geq P\beta^{2}\left(\Delta -c_{n}-Pe\right)/e$. According to the assumption $\Delta >c_{n}+Pe$, we know $D_{2}\left(p,s\right)>0$, and thus (iii) holds. (2) Consider *Model R: The remanufacturing strategy* We know $\partial^{2}\Pi_{R}/\partial p^{2}=-2<0$ and $\partial^{2}\Pi_{R}/\partial \tau^{2}=-2A<0$, since A>0. Therefore, $\Pi_{R}$ is concave in p and τ only if $D_{2}\left(p,\tau \right)=\left|\begin{array}{cc} -2 & -Pb-a\\ -Pb-a & -2A \end{array}\right|=4A-{\left(Pb+a\right)}^{2}>0$. To make sure the optimal solution of τ is between 0 and 1, we impose the condition $\partial \Pi_{R}/{\left.\partial \tau \right|}_{\tau =1}\leq 0$, from which we can obtain the following result:

(A2) $$4A\geq \left(Pb+a\right)\left(\Delta -c_{n}-Pe\right)+{\left(Pb+a\right)}^{2}$$

Therefore, $D_{2}\left(p,\tau \right)>0$ holds from the previous condition. (3) Consider *Model I&R: The strategy involving both investment and remanufacturing* The Hessian matrix of $\Pi_{I\&R}$ is $H\left(p,\tau ,s\right)=\left(\begin{array}{ccc} -2 & -Pb-a & -P\beta \\ -Pb-a & -2A & 0\\ -P\beta & 0 & -2B \end{array}\right)$. Therefore, the second derivatives for p,τ,s are all negative; the second order principal minor of $H\left(p,\tau ,s\right)$ is $D_{2}\left(p,\tau ,s\right)=4A-{\left(Pb+a\right)}^{2}$, which is positive from the condition ($\partial \Pi_{R}/{\left.\partial \tau \right|}_{\tau =1}\leq 0$) of model R; and the third order principal minor of $H\left(p,\tau ,s\right)$ is $D_{3}\left(p,\tau ,s\right)=-8AB+2B{\left(Pb+a\right)}^{2}+2AP^{2}\beta^{2}$. Thus, $\Pi_{I\&R}$ is concave in p,τ,s only if $D_{3}\left(p,\tau ,s\right)<0$ holds. To make sure both the optimal solutions of τ and s are on their domains, we simultaneously impose the conditions as follows:

$$\left\{\begin{array}{c} \partial \Pi_{I\&R}/{\left.\partial \tau \right|}_{\tau =1}\leq 0\\ \partial \Pi_{I\&R}/{\left.\partial s\right|}_{s=e/\beta }\leq 0 \end{array}\right.\;$$

From the condition $\partial \Pi_{I\&R}/{\left.\partial \tau \right|}_{\tau =1}\leq 0$, we obtain

(A3) $$B\geq AP^{2}\beta^{2}/\left(4A-\left(Pb+a\right)\left(\Delta -c_{n}-Pe\right)-{\left(Pb+a\right)}^{2}\right)$$

From the condition $\partial \Pi_{I\&R}/{\left.\partial s\right|}_{s=e/\beta }\leq 0$, we obtain

(A4) $$B\geq AP^{2}\beta^{2}\left(\Delta -c_{n}\right)/e\left(4A-{\left(Pb+a\right)}^{2}\right)$$

From (A4), $D_{3}\left(p,\tau ,s\right)\leq -2AP^{2}\beta^{2}\left(\Delta -c_{n}-Pe\right)/e<0$. Therefore, $\Pi_{I\&R}$ is concave in p,τ,s. Comparing (A4) and (A1), we know that if (A4) holds, then (A1) must hold, because $\frac{P\beta^{2}\left(\Delta -c_{n}\right)}{4e}-\frac{AP^{2}\beta^{2}\left(\Delta -c_{n}\right)}{e\left(4A-{\left(Pb+a\right)}^{2}\right)}=-\frac{P\beta^{2}\left(\Delta -c_{n}\right){\left(Pb+a\right)}^{2}}{4e\left(4A-{\left(Pb+a\right)}^{2}\right)}<0$. Comparing (A3) and (A2), we know that if (A3) holds, then (A2) must hold, because from (A3), we have $4A\geq \left(Pb+a\right)\left(\Delta -c_{n}-Pe\right)+{\left(Pb+a\right)}^{2}+AP^{2}\beta^{2}/B$, from which we know (A2) must hold. Comparing (A4) and (A3), we know that if (A3) holds, then (A4) must hold, because

$$\begin{array}{c} \frac{AP^{2}\beta^{2}}{4A-\left(Pb+a\right)\left(\Delta -c_{n}-Pe\right)-{\left(Pb+a\right)}^{2}}-\frac{AP^{2}\beta^{2}\left(\Delta -c_{n}\right)}{e\left(4A-{\left(Pb+a\right)}^{2}\right)}\\ =\frac{AP\beta^{2}\left(\Delta -c_{n}-Pe\right)\left(\left(Pb+a\right)\left(\Delta -c_{n}\right)+4A-{\left(Pb+a\right)}^{2}\right)}{e\left(4A-\left(Pb+a\right)\left(\Delta -c_{n}-Pe\right)-{\left(Pb+a\right)}^{2}\right)\left(4A-{\left(Pb+a\right)}^{2}\right)}>0 \end{array}$$

Therefore, if (A3) holds, then (A1), (A2), and (A4) must hold, which means that $s^{*I}$, $s^{*I\&R}$ are all between 0 and e/β. $\tau^{*R}$, $\tau^{*I\&R}$ are all between 0 and 1. □

**Table A1:** Comparison of the manufacturer’s operational models with and without cap-and-trade.

Optimal Decisions and Performance	Model W	Model B
$\Pi^{*}$	$\frac{{\left(\Delta -c_{n}\right)}^{2}}{4}$	$PC+\frac{{\left(\Delta -c_{n}-Pe\right)}^{2}}{4}$
$E^{*}$	$\frac{e\left(\Delta -c_{n}\right)}{2}$	$\frac{e\left(\Delta -c_{n}-Pe\right)}{2}$
$q^{*}$	$\frac{\Delta -c_{n}}{2}$	$\frac{\Delta -c_{n}-Pe}{2}$
$p^{*}$	$\frac{\Delta +c_{n}}{2}$	$\frac{\Delta +c_{n}+Pe}{2}$

**Proof** **of** **Proposition** **1.** We can easily find the phenomenon from Table A1 that the production quantity, the selling price, and the carbon emissions are affected by the carbon price and carbon emission of the product, while they are not affected by the cap in Model B.

Comparing the optimal profits of Model B and Model W, we have $\Pi^{*B}-\Pi^{*W}=P\left(C-e\left(2\Delta -2c_{n}-Pe\right)/4\right)$

Therefore, when $4C>e\left(2\Delta -2c_{n}-Pe\right)$, we have $\Pi^{*B}>\Pi^{*W}$.

Equivalently, $T=E-C<-Pe^{2}/4$, which means the manufacturer’s cap is adequate for her production and she even has some surplus allowances that can be sold in the carbon emissions market to make profits. □

**Table A2:** Comparison of the manufacturer’s carbon abatement models with cap-and-trade.

Optimal Decisions and Performance	Model I	Model R	Model I&R
$\Pi^{*}$	$PC+\frac{B{\left(\Delta -c_{n}-Pe\right)}^{2}}{4B-P^{2}\beta^{2}}$	$PC+\frac{A{\left(\Delta -c_{n}-Pe\right)}^{2}}{4A-{\left(Pb+a\right)}^{2}}$	$PC+\frac{AB{\left(\Delta -c_{n}-Pe\right)}^{2}}{A\left(4B-P^{2}\beta^{2}\right)-B{\left(Pb+a\right)}^{2}}$
$E^{*}$	$\begin{array}{c} \frac{B\left(Be-2P\beta^{2}\left(\Delta -c_{n}\right)\right)}{{\left(4B-P^{2}\beta^{2}\right)}^{2}}\\ \cdot \;\left(\Delta -c_{n}-Pe\right) \end{array}$	$\begin{array}{c} \frac{2A\left(e\left(4A-Pab-a^{2}\right)-b\left(\Delta -c_{n}\right)\left(Pb+a\right)\right)}{{\left(4A-{\left(Pb+a\right)}^{2}\right)}^{2}}\\ \cdot \;\left(\Delta -c_{n}-Pe\right) \end{array}$	$\begin{array}{c} \frac{2AB\left(Be\left(4A-Pb-a^{2}\right)-\left(\Delta -c_{n}\right)\left(AP\beta^{2}+Bb\left(Pb+a\right)\right)\right)}{{\left(A\left(4B-P^{2}\beta^{2}\right)-B{\left(Pb+a\right)}^{2}\right)}^{2}}\\ \cdot \;\left(\Delta -c_{n}-Pe\right) \end{array}$
$q^{*}$	$\frac{2B\left(\Delta -c_{n}-Pe\right)}{4B-P^{2}\beta^{2}}$	$\frac{2A\left(\Delta -c_{n}-Pe\right)}{4A-{\left(Pb+a\right)}^{2}}$	$\frac{2AB\left(\Delta -c_{n}-Pe\right)}{A\left(4B-P^{2}\beta^{2}\right)-B{\left(Pb+a\right)}^{2}}$
$p^{*}$	$\frac{2B\left(\Delta +c_{n}+Pe\right)-\Delta P^{2}\beta^{2}}{4B-P^{2}\beta^{2}}$	$\frac{2A\left(\Delta +c_{n}+Pe\right)-\Delta {\left(Pb+a\right)}^{2}}{4A-{\left(Pb+a\right)}^{2}}$	$\frac{2AB\left(\Delta +c_{n}+Pe\right)-\Delta B{\left(Pb+a\right)}^{2}-\Delta AP^{2}\beta^{2}}{A\left(4B-P^{2}\beta^{2}\right)-B{\left(Pb+a\right)}^{2}}$
$s^{*}$	$\frac{P\beta \left(\Delta -c_{n}-Pe\right)}{4B-P^{2}\beta^{2}}$	N/A	$\frac{AP\beta \left(\Delta -c_{n}-Pe\right)}{A\left(4B-P^{2}\beta^{2}\right)-B{\left(Pb+a\right)}^{2}}$
$\tau^{*}$	N/A	$\frac{\left(Pb+a\right)\left(\Delta -c_{n}-Pe\right)}{4A-{\left(Pb+a\right)}^{2}}$	$\frac{B\left(Pb+a\right)\left(\Delta -c_{n}-Pe\right)}{A\left(4B-P^{2}\beta^{2}\right)-B{\left(Pb+a\right)}^{2}}$

**Proof** **of** **Proposition** **2.** The proof of Proposition 2 is composed of two parts.

(i) First, we prove $q^{*I\&R}>q^{*R},q^{*I}>q^{*B}$ through simple algebra.
  $q^{*B}=\frac{\Delta -c_{n}-Pe}{2}=\frac{2B\left(\Delta -c_{n}-Pe\right)}{4B}<\frac{2B\left(\Delta -c_{n}-Pe\right)}{4B-P^{2}\beta^{2}}=q^{*I}$. Similarly, we can prove $q^{*R}>q^{*B}$, $q^{*I\&R}>q^{*I}$, and $q^{*I\&R}>q^{*R}$.
(ii) Second, we prove the relationship between $q^{*I}$ and $q^{*R}$.
  $q^{*R}-q^{*I}=\frac{\left(-2AP^{2}\beta^{2}+2B{\left(Pb+a\right)}^{2}\right)\left(\Delta -c_{n}-Pe\right)}{\left(4B-P^{2}\beta^{2}\right)\left(4A-{\left(Pb+a\right)}^{2}\right)}$. From the assumption $\Delta >c_{n}+Pe$, we know $\Delta -c_{n}-Pe>0$. From (A1) and (A2), we know $\left(4B-P^{2}\beta^{2}\right)\left(4A-{\left(Pb+a\right)}^{2}\right)>0$. Therefore, when ${\left(Pb+a\right)}^{2}/A>P^{2}\beta^{2}/B$, we have $q^{*R}>q^{*I}$, otherwise, $q^{*R}<q^{*I}$.
  Because the ranking of the production quantity holds, the ranking of the price also holds. □

**Proof** **of** **Proposition** **3.** The value of $s^{*}$ (the sustainability level) and $\tau^{*}$ (the return rate of used products) in Table A2 can be easily compared and is trivial to prove. □

**Proof** **of** **Proposition** **4.** In Table A2, it is obvious that $\Pi^{*I}>\Pi^{*B}$, $\Pi^{*R}>\Pi^{*B}$, $\Pi^{*I\&R}>\Pi^{*I}$, and $\Pi^{*I\&R}>\Pi^{*R}$. For the relationship between $\Pi^{*I}$ and $\Pi^{*R}$, after simplification, we have $\Pi^{*R}-\Pi^{*I}=\frac{\left(-AP^{2}\beta^{2}+B{\left(Pb+a\right)}^{2}\right){\left(\Delta -c_{n}-Pe\right)}^{2}}{\left(4B-P^{2}\beta^{2}\right)\left(4A-{\left(Pb+a\right)}^{2}\right)}$. Therefore, when ${\left(Pb+a\right)}^{2}/A>P^{2}\beta^{2}/B$, we have $\Pi^{*R}>\Pi^{*I}$; otherwise, $\Pi^{*R}<\Pi^{*I}$. □

**Proof** **of** **Proposition** **5.** The proof of Proposition 5 is similar to the proof of Proposition 2, and we omit it. □

**Proof** **of** **Proposition** **6.** The value of $s^{*CI}$, $s^{*CI\&CR}$ (the sustainability level) and $\tau^{*CR}$, $\tau^{*CI\&CR}$ (the return rate of used products) can be easily compared and is trivial to prove. □

**Proof** **of** **Proposition** **7.** We divide the proof of Proposition 7 into three parts.

(i) We identify the conditions under which the CI strategy, the CR strategy, and the CI&CR strategy are superior to the CB strategy.
  $$\Pi^{*CI}-\Pi^{*CB}=\frac{P^{2}\beta^{2}X_{1}^{2}X_{2}}{X_{3}^{2}{\left(d_{1}d_{2}-4\right)}^{2}}$$
  where $X_{1}=\left(\left(\alpha -1\right)\Delta -c_{c}-Pe\right)d_{1}-\left(d_{1}d_{2}-2\right)\left(Pe+c_{n}\right)-2\Delta \alpha$,$X_{2}=\left(4B-P^{2}\beta^{2}\right){\left(d_{1}d_{2}-2\right)}^{2}-Bd_{1}^{2}d_{2}^{2}$, and $X_{3}=P^{2}\beta^{2}\left(2-d_{1}d_{2}\right)-2B\left(d_{1}d_{2}-4\right)$.
  Therefore, if $X_{2}>0$, then $\Pi^{*CI}>\Pi^{*CB}$, which is $\left(4B-P^{2}\beta^{2}\right){\left(d_{1}d_{2}-2\right)}^{2}-Bd_{1}^{2}d_{2}^{2}>0$.
  After simplification, we have $P^{2}\beta^{2}/B<4-d_{1}^{2}d_{2}^{2}/{\left(d_{1}d_{2}-2\right)}^{2}$.
  $$\Pi^{*CR}-\Pi^{*CB}=\frac{{\left(Pb+a\right)}^{2}Y_{1}^{2}Y_{2}}{Y_{3}^{2}{\left(d_{1}d_{2}-4\right)}^{2}}$$
  where $Y_{1}=X_{1}$, $Y_{2}=\left(4A-{\left(Pb+a\right)}^{2}\right){\left(d_{1}d_{2}-2\right)}^{2}-Ad_{1}^{2}d_{2}^{2}$, and $Y_{3}={\left(Pb+a\right)}^{2}\left(2-d_{1}d_{2}\right)-2A\left(d_{1}d_{2}-4\right)$.
  Similarly, if $Y_{3}>0$, then $\Pi^{*CR}>\Pi^{*CB}$. After simplification, we have ${\left(Pb+a\right)}^{2}/A<4-d_{1}^{2}d_{2}^{2}/{\left(d_{1}d_{2}-2\right)}^{2}$.
  $$\Pi^{*CI\&CR}-\Pi^{*CB}=\frac{\left(AP^{2}\beta^{2}+B{\left(Pb+a\right)}^{2}\right)Z_{1}^{2}Z_{2}}{Z_{3}^{2}{\left(d_{1}d_{2}-4\right)}^{2}}$$
  where $Z_{1}=X_{1}$, $Z_{2}=\left(4AB-AP^{2}\beta^{2}-B{\left(Pb+a\right)}^{2}\right){\left(d_{1}d_{2}-2\right)}^{2}-ABd_{1}^{2}d_{2}^{2}$, and $Z_{3}=\left(d_{1}d_{2}-2\right)\left(B{\left(Pb+a\right)}^{2}+AP^{2}\beta^{2}\right)-2AB\left(d_{1}d_{2}-4\right)$.
  Similarly, if $Z_{3}>0$, then $\Pi^{*CI\&CR}>\Pi^{*CB}$. After simplification, we have the following condition:
  $$P^{2}\beta^{2}/B+{\left(Pb+a\right)}^{2}/A<4-d_{1}^{2}d_{2}^{2}/{\left(d_{1}d_{2}-2\right)}^{2}$$
(ii) We compare the CI strategy with the CR strategy.
  $$\Pi^{*CR}-\Pi^{*CI}=-\frac{W_{1}^{2}W_{2}W_{3}}{W_{4}^{2}W_{5}{}^{2}}$$
  where $W_{1}=X_{1}$, $W_{2}=AP^{2}\beta^{2}-B{\left(Pb+a\right)}^{2}$,$W_{3}=\left(4B-AP^{2}\beta^{2}\right)\left(4A-{\left(Pb+a\right)}^{2}\right){\left(d_{1}d_{2}-2\right)}^{2}-4ABd_{1}^{2}d_{2}^{2}$, $W_{4}=Y_{3}$, and $W_{5}=X_{3}$.
  We can easily obtain the conditions by simple algebra, which is shown in Proposition 7(iii).
(iii) We identify the conditions under which the CI&CR strategy is superior to the CI strategy and the CR strategy.
  $$\Pi^{*CI\&CR}-\Pi^{*CI}=\frac{B{\left(Pb+a\right)}^{2}U_{1}^{2}U_{2}}{U_{3}^{2}U_{4}^{2}}$$
  where $U_{1}=X_{1}$, $U_{3}=Z_{4}$, $U_{4}=X_{3}$, and $U_{2}=\left(4AB-AP^{2}\beta^{2}-B{\left(Pb+a\right)}^{2}\right)\left(4B-AP^{2}\beta^{2}\right){\left(d_{1}d_{2}-2\right)}^{2}-4AB^{2}d_{1}^{2}d_{2}^{2}$.

Therefore, when $\Pi^{*CI\&CR}>\Pi^{*CI}$, we have the condition:

$$P^{2}\beta^{2}/B+{\left(Pb+a\right)}^{2}/A<4-4m/\left(4-P^{2}\beta^{2}/B\right)$$

where $m=d_{1}^{2}d_{2}^{2}/{\left(d_{1}d_{2}-2\right)}^{2}$.

$$\Pi^{*CI\&CR}-\Pi^{*CR}=\frac{AP^{2}\beta^{2}V_{1}^{2}V_{2}}{V_{3}^{2}V_{4}^{2}}$$

where $V_{1}=X_{1}$, $V_{3}=Z_{4}$, $V_{4}=Y_{3}$, and $V_{2}=\left(4AB-AP^{2}\beta^{2}-B{\left(Pb+a\right)}^{2}\right)\left(4A-{\left(Pb+a\right)}^{2}\right){\left(d_{1}d_{2}-2\right)}^{2}-4A^{2}Bd_{1}^{2}d_{2}^{2}$. Therefore, when $\Pi^{*CI\&CR}>\Pi^{*CR}$, we have the condition:

$$P^{2}\beta^{2}/B+{\left(Pb+a\right)}^{2}/A<4-4m/\left(4-{\left(Pb+a\right)}^{2}/A\right)$$

where $m=d_{1}^{2}d_{2}^{2}/{\left(d_{1}d_{2}-2\right)}^{2}$. Thus, in summary, if $SR_{I}+SR_{R}<\min\left\{4-4m/\left(4-SR_{I}\right),4-4m/\left(4-SR_{R}\right)\right\}$, $\Pi_{M}^{*CI\&CR}>\Pi_{M}^{*CR},\Pi_{M}^{*CI}$, where $SR_{I}=P^{2}\beta^{2}/B$, $SR_{R}={\left(Pb+a\right)}^{2}/A$, $m=d_{1}^{2}d_{2}^{2}/{\left(d_{1}d_{2}-2\right)}^{2}$. By simple algebra, we can easily prove $\Pi^{*CI\&CR}>\Pi^{*CB}$ holds under this condition. □
